# Dynamic optoelectric trapping and deposition of multiwalled carbon nanotubes

**DOI:** 10.1038/micronano.2016.5

**Published:** 2016-03-24

**Authors:** Avanish Mishra, Katherine Clayton, Vanessa Velasco, Stuart J. Williams, Steven T. Wereley

**Affiliations:** 1 Birck Nanotechnology Center, School of Mechanical Engineering, Purdue University, West Lafayette, IN 47907, USA; 2 Department of Mechanical Engineering, University of Louisville, KY 40292, USA

**Keywords:** carbon nanotube, deposition, optoelectrical tweezers, patterning, trapping

## Abstract

In the path toward the realization of carbon nanotube (CNT)-driven electronics and sensors, the ability to precisely position CNTs at well-defined locations remains a significant roadblock. Highly complex CNT-based bottom–up structures can be synthesized if there is a method to accurately trap and place these nanotubes. In this study, we demonstrate that the rapid electrokinetic patterning (REP) technique can accomplish these tasks. By using laser-induced alternating current (AC) electrothermal flow and particle–electrode forces, REP can collect and maneuver a wide range of vertically aligned multiwalled CNTs (from a single nanotube to over 100 nanotubes) on an electrode surface. In addition, these trapped nanotubes can be electrophoretically deposited at any desired location onto the electrode surface. Apart from active control of the position of these deposited nanotubes, the number of CNTs in a REP trap can also be dynamically tuned by changing the AC frequency or by adjusting the concentration of the dispersed nanotubes. On the basis of a calculation of the stiffness of the REP trap, we found an upper limit of the manipulation speed, beyond which CNTs fall out of the REP trap. This peak manipulation speed is found to be dependent on the electrothermal flow velocity, which can be varied by changing the strength of the AC electric field.

## Introduction

Owing to their extraordinary chemical, electrical and mechanical properties, carbon nanotubes (CNTs) have been widely explored as transistors, ion^1^ and gas sensors^2,3^, biosensors^4^ and field emission devices^5,6^. These applications often require individual CNTs or groups of CNTs to be positioned on an electrode surface^7–9^. Presently, patterned CNTs are fabricated using two sets of methods: the conventional direct-growth chemical vapor deposition (CVD) methods and the post-growth methods (where CNTs are deposited from a solution onto a substrate^10^). Being free of growth constraints, such as temperature or substrate material, post-growth methods offer versatility and can be used with purified metallic or semiconducting CNTs^11,12^. Alternatively, direct-growth CVD methods produce a mixture of metallic and semiconducting CNTs and use high-temperature processes, which limit their applicability in microelectronic device fabrication^13^. Thus, various post-growth methods, such as Langmuir–Blodgett^14^, Langmuir–Schaefer^15^, contact printing^10^, chemical assembly^16^, evaporation-driven self-assembly^17^, electrophoretic deposition^18,19^, dielectrophoresis^13,20,21^, optical tweezers^22–26^ and optoelectronic tweezers (OET)^27^, have been proposed for the alignment, assembly and trapping of CNTs at room temperature.

Among these methods, both optical tweezers^22–26^ and optoelectronic tweezers (OET)^27^ invoke special interest owing to their ability to dynamically trap and translate CNTs via optical patterns. These approaches allow for highly controllable placement. However, a few limitations restrict the widespread application of both of these methods. For example, optical tweezers have been used to trap bundles of single-wall carbon nanotubes. However, trapping single nanotubes remains a challenging task^22–26^. In contrast, OET can trap individual nanotubes, but this technique requires a photoconductive substrate, limiting its implementation in device fabrication. In this study, we present a post-growth optoelectric method, referred to as rapid electrokinetic patterning (REP) that can trap both a single CNT and hundreds of vertically oriented CNTs by focusing a laser beam onto an electrode surface in the presence of a uniform alternating current (AC) electric field^28–30^. Unlike OET, REP does not require a photoconductive substrate and is more flexible than optical tweezers, as it can trap either individual or multiple nanotubes.

We demonstrate the potential of REP by showing reversible trapping and manipulation of multiwalled carbon nanotubes (MWCNTs), which are observed via dark-field microscopy. Apart from reversible trapping, the trapped MWCNTs are electrophoretically deposited^9,18,19,31^, allowing us to rapidly assemble a desired number of vertically oriented nanoelectrodes at a selected location on the electrode surface.

### Operational principle

**Figure 1:**
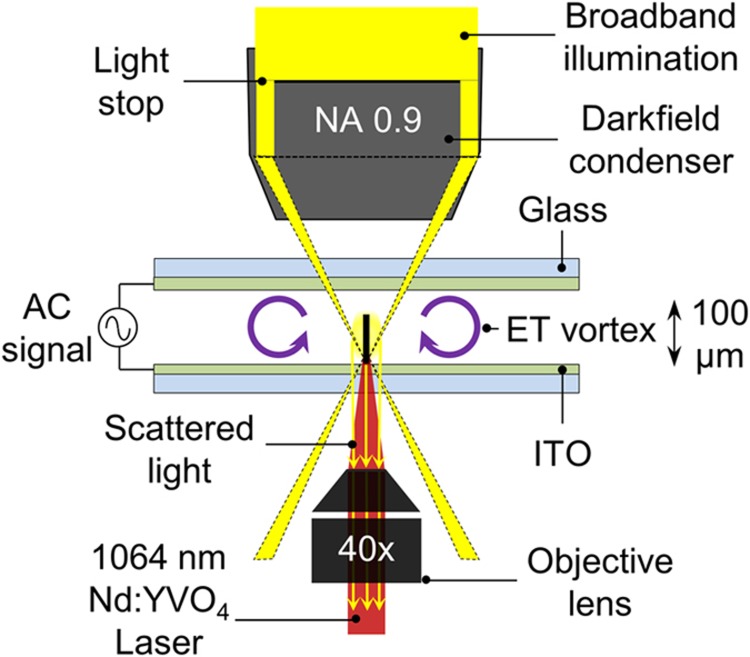
Schematic of the rapid electrokinetic patterning (REP) setup. A REP chip is comprised of two indium tin oxide (ITO)-coated glass substrates that are separated by a 100-μm thick spacer. An aqueous dispersion of multiwalled carbon nanotubes (MWCNTs) is sandwiched between the ITO electrodes. For trapping the nanotubes, an infrared (1064 nm) laser beam is projected on the bottom electrode in the presence of an AC electric field. MWCNTs are imaged under a dark-field microscope equipped with an air dark-field condenser and a 40× objective lens. Supplementary Information for this article can be found on the *Microsystems & Nanoengineering* website (http://www.nature.com/micronano).

A REP chip is composed of two transparent featureless indium tin oxide (ITO)-coated parallel-plate electrodes separated by a 100-μm thick spacer (Figure 1). The space between electrodes is filled with an aqueous particle-laden solution. The REP mechanism uses a combination of electrokinetic effects, such as electroorientation^32^, laser-induced electrothermal flow^33,34^ and particle–electrode interactions^35^, for reversible particle trapping. In the presence of an AC electric field, particles in the solution are polarized, causing them to experience dipole–dipole repulsion from one another, orthogonal to the direction of the electric field^30,32^. Focusing an infrared laser beam on the bottom electrode surface heats the irradiated region owing to partial absorption of the radiation by the 700-nm thick ITO layer. The laser-heated region warms the liquid in its vicinity, resulting in a temperature gradient, which in turn produces gradients in the electrical conductivity and permittivity of the liquid. This variance, coupled with the uniform AC electric field, generates a toroidal electrothermal vortex that sweeps particles into a stagnation zone located at the center of the vortex on the electrode surface^33^. Particles are trapped in this region with the assistance of particle–electrode interactions^36^. The trapped particles can be relocated relative to the electrode surface by translating the REP chip on a microscope stage, while keeping the laser spot fixed. The stage displacement creates an electrothermal vortex at a new position on the REP chip within a fraction of a second^37^. Consequently, the trapped particles are swept to the new spot. Disabling the laser and electric field releases the captured particles from the trap.

## Materials and methods

### REP chip preparation

To prepare a REP chip, two 700-nm ITO-coated glass (SPI Supplies Inc., West Chester, PA, USA) pieces that measured 25.4 mm×25.4 mm were selected. Two 1-mm holes were drilled in one of the ITO glass pieces to provide inlet and outlet ports for the MWCNT solution. Both the ITO glass pieces were ultrasonicated for 3 min each in acetone, isopropanol and methanol. The solvent-cleaned glass pieces were rinsed with deionized water and then dried with nitrogen gas. Next, two layers of 50-μm thick double-sided tape were cut in the shape of the channel geometry and then sandwiched between the two ITO cover slips. The inlet and outlet ports were sealed with 1.6-mm thick adhesive silicone rubber pads^35^ (CS Hyde Company, Lake Villa, IL, USA), through which polyethylene tubes were inserted for supplying the MWCNT dispersion. Two strips of copper tape were adhered to the exposed ITO to provide electrical connections for the application of the AC electric field.

### Apparatus

A continuous wave 1064-nm Nd:YVO_4_ laser was coupled into an inverted Nikon TE2000U microscope (Nikon Instruments Inc., Melville, NY, USA). MWCNTs were observed via dark-field microscopy, which was performed using a dark-field air condenser (0.80–0.95 numerical aperture (NA)) and a Nikon 40× ELWD objective (0.6 NA). The MWCNTs are visible under dark-field microscopy because of Rayleigh scattering. The 40× objective was additionally used to focus a slightly diverging laser beam 25 μm above the focal plane of the objective lens. This approach resulted in a beam waist radius of 15 μm within the imaging plane^38^. Dark-field images of MWCNTs were recorded using a 16-bit grayscale PCO.1600 CCD camera (PCO AG, Kelheim, Germany) controlled with Camware^®^ software (PCO AG, Kelheim, Germany).

A motorized microscope stage (Prior Scientific, Inc., Rockland, MA, USA) was used for translating the REP chips to demonstrate MWCNT manipulation. An AC electric field was applied between the electrodes using a function generator (Model 625A, Berkeley Nucleonics Corporation, San Rafael, CA, USA). The applied electric field and frequency were monitored using an oscilloscope (Model 54610B, Agilent Technologies, Santa Clara, CA, USA). For REP trapping, the laser power was kept at 20 mW, while the electric field and frequency were varied in the ranges of 100–140 kV m^−1^ and 10–100 kHz, respectively. In this manuscript, the electric field magnitudes are reported as peak-to-peak values.

### Carbon nanotube sample preparation

An aqueous dispersion of MWCNTs (3.0% by weight) was purchased from US Research Nanomaterials, Inc. (Houston, TX, USA). The outer diameter and length of the MWCNTs were in the ranges of 50 to 80 nm and 10 to 20 μm, respectively. Aliquots of 100 μl of the stock solution were diluted 50-fold in deionized water. One milliliter of the diluted solution was ultrasonicated for 1 min, followed by centrifugation for 15 min at 12,000×g to remove large bundles of nanotubes, which were collected in a pellet. After centrifugation, the supernatant samples were inspected using dark-field microscopy for nanotube bundles. The samples containing particles circular in appearance and radii >2.5 μm were discarded. The supernatant solution was further diluted with deionized water to achieve CNT concentrations of 1.3×10^7^ CNTs per ml and 1.6×10^8^ CNTs per ml to demonstrate REP trapping of a single MWCNT and an ensemble of MWCNTs, respectively. For the purpose of demonstrating control over the number of nanotubes contained in a REP trap, two CNT concentrations of 2.3×10^8^ CNTs per ml and 7.8×10^7^ CNTs per ml were used. The conductivity of the supernatant was measured to be 0.37±0.04 mS m^−1^ using a Zetasizer Nano ZS (Malvern Instruments Ltd, Malvern, UK).

### Particle tracking velocimetry and particle counting

The velocity profile of a single nanotube moving into the REP trap was measured to determine the peak manipulation velocity. The images of the nanotubes were recorded at the following trapping conditions: a laser power of 20 mW, electric field strengths of 100 and 140 kV m^−1^, and an AC frequency of 15 kHz. The nanotubes were tracked using the Manual Tracking plugin in the image analysis software, ImageJ (http://rsb.info.nih.gov/ij/). The number of nanotubes collected in the REP trap was determined using the Analyze Particles tool in ImageJ.

## Results and discussion

### Trapping and manipulation of a single MWCNT

**Figure 2:**
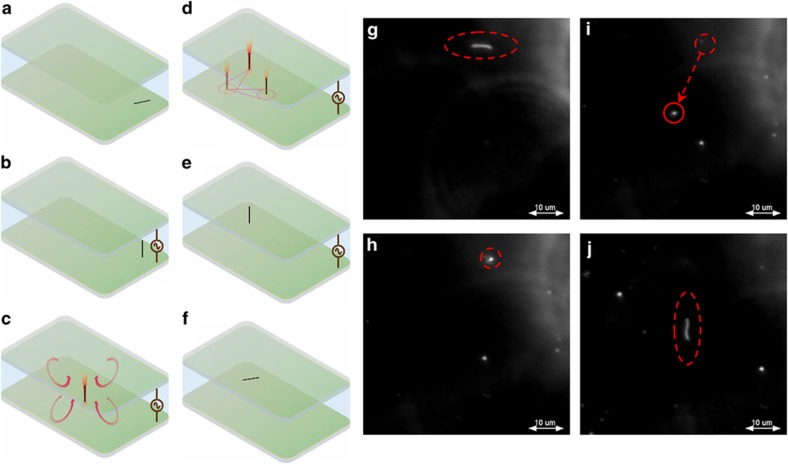
REP trapping and manipulation of a single MWCNT. (**a**–**f**) Schematics illustrating the steps of the REP trapping process. (**a**) In the absence of the AC electric field and the laser beam, the MWCNT is unaligned. (**b**) Application of the electric field (140 kV m^−1^, 55 kHz) aligns the nanotube parallel to the field. (**c**) Projecting the laser beam in the presence of the electric field creates an electrothermal vortex that traps the vertically oriented nanotube at the center of the vortex. (**d**) The trapped nanotube is manipulated by moving the REP chip, while keeping the laser at a fixed spot. (**e**) Switching the laser off stops the electrothermal flow, and the nanotube remains in a vertical orientation at the transported position. (**f**) Nanotube loses its vertical orientation upon removal of the field. (**g** and **h**) Dark-field images corresponding to the schematic diagrams in **a**–**c** and **f**. (**g**) A dark-field image of an unaligned nanotube. (**h**) An image of the nanotube in a vertical orientation under the application of the electric field. (**i**) An image of the trapped nanotube in the presence of the electric field and laser. (**j**) An image of the released nanotube.

To demonstrate flexible trapping and manipulation of a single nanotube in a REP trap, a diluted aqueous dispersion of MWCNTs (1.3×10^7^ particles per ml) was used. Figure 2 and Movie M1 in the Supplementary Information show the trapping process. In the absence of an electric field and a laser, a single, untrapped MWCNT sampled various orientations, as shown in Figures 2a and g. The application of an AC electric field (140 kV m^−1^, 55 kHz) resulted in an induction of a dipole moment around the nanotube. The polarized nanotube experienced an aligning torque that oriented it in the vertical direction, parallel to the applied field within 1 s (Figures 2b and h).

Focusing a 20-mW laser beam on the bottom electrode surface resulted in the trapping of the nanotube at the center of the electrothermal vortex on the bottom electrode (Figures 2c and i). The trapped nanotube was manipulated by moving the REP chip on a motorized sample stage while keeping the laser spot fixed (Figure 2d). After REP manipulation, the nanotube was released from the trap by switching-off the laser beam and electric field. Closing the laser shutter stopped the electrothermal flow (Figure 2e), and the removal of the electric field caused the MWCNT to lose its orientation (Figures 2f and j). These observations demonstrate that REP can be used to reversibly trap and manipulate a single MWCNT.

Considering a REP trap as a potential well with a constant stiffness, the overdamped equation of the motion of the MWCNT under manipulation in the *x* direction can be expressed by Equation (1)^39–42^.

(1)β(x˙−x˙drive)+αxx=F(t)

Owing to the ultralow Reynolds number (~10^−6^) of the flow around the MWCNT, inertial forces are negligible compared with viscous forces. In Equation (1), ${\dot{x}}_{\mathrm{drive}}$ is the microscope stage velocity in the *x* direction, *β* is the hydrodynamic drag coefficient of the MWCNT, *x* is the position of the MWCNT from its equilibrium position, *α*_*x*_ is the stiffness of the trap in the *x* direction, and *F*(*t*) is the random time-dependent force due to thermal fluctuations. A similar equation can be written for the nanotube manipulation in the *y* direction. To estimate the trap stiffness, we used the Equipartition theorem, which states that^39^

(2)12kbT=12αx〈x2〉

(3)12kbT=12αy〈y2〉

where *α*_*y*_ is the trap stiffness in the *y* direction, *k*_b_ is the Boltzmann constant, *T* is the temperature of the medium (293±1 K), and 〈*x*^2^〉 and 〈*y*^2^〉 are the variance in the position of the MWCNT in the *x* and *y* directions, respectively. Therefore, the REP trap stiffness can be determined by estimating the position variance from the microscopy images (see the Supplementary Information file for further discussion). For a 2-μm long MWCNT, 〈*x*^2^〉 and 〈*y*^2^〉 are found to be 0.093±0.002 μm^2^ and 0.104±0.002 μm^2^, respectively. Consequently, the trap stiffness in the *x* and *y* directions were calculated to be 43.3±0.9 and 38.6±0.8 fN μm^−1^, respectively. The REP trap stiffness values are same order of magnitude as the trap stiffness reported for the trapping of an MWCNT by OET^27^. The small difference in the *α*_*x*_ and *α*_*y*_ values can be attributed to a slight variation in the velocity profile of the electrothermal flow around the axis of symmetry.

**Figure 3:**
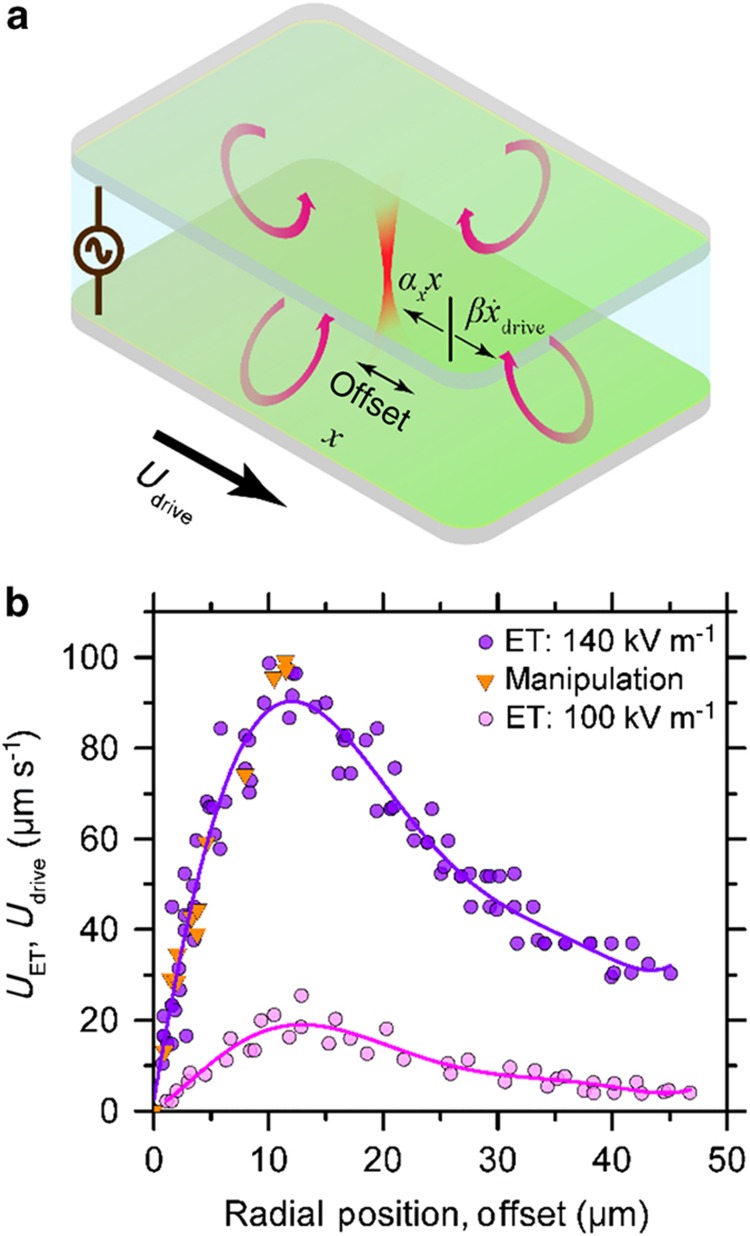
(**a**) Moving a REP chip with velocity $U_{\mathrm{drive}}$ ($={\dot{x}}_{\mathrm{drive}}$) causes the nanotube to acquire an offset *x* with respect to the center of the laser. At this new position, the drag force on the nanotube $\beta {\dot{x}}_{drive}$ is equal and opposite to the trapping force *α*_*x*_*x*. (**b**) Velocity of a nanotube moving into a REP trap as a function of radial position from the center of the trap. The electric field was varied from 140 to 100 kV m^−1^ while the laser power and AC frequency were kept constant at 20 mW and 15 kHz, respectively. Solid orange triangles represent the REP chip velocity and the corresponding radial offset of a nanotube from the trap center.

During REP manipulation, the nanotube acquires an offset with respect to the trap center. The effect of manipulation speed on the offset can be explained by the rearranged form of Equation (1), $\beta {\dot{x}}_{\mathrm{drive}}-\alpha_{x}x=\beta \dot{x}-F(t)$. At an offset *x*, the drag force on the nanotube owing to the trapping chamber movement $\beta {\dot{x}}_{\mathrm{drive}}$ is equal and opposite to the trapping force *α*_*x*_*x*. Increasing the manipulation velocity ${\dot{x}}_{\mathrm{drive}}$ causes the nanotube to move to a higher offset, where an increased drag force is balanced by a higher trapping force, as shown in Figure 3b. Beyond a peak manipulation velocity, the nanotube cannot be translated while remaining in the REP trap as the drag force $\beta {\dot{x}}_{\mathrm{drive}}$ becomes greater than the maximum trapping force *α*_*x*_*x*_max_. This process results in a net force on the nanotube, causing it to move away from the trap center at a velocity of $\dot{x}$. As a result, the nanotube falls out of the REP trap. For the 140 kV m^−1^ AC electric field, the peak manipulation velocity and the corresponding offset *x*_max_ were experimentally determined to be ~95 μm s^−1^ and ~11.5 μm, respectively. Following the treatment proposed by Pauzauskie *et al.*^27^, the drag force on the MWCNT at the peak manipulation velocity is found to be 503 fN, which is close to the value of the expected maximum trapping force *α*_*x*_*x*_max_ (~498 fN).

The trapping force in REP is caused by the radially inward flow of the electrothermal vortex. The radially inward velocity of the electrothermal flow increases with radial position until a peak velocity is reached (Figure 3b). Hence, the trapping force will achieve a maximum value corresponding to the position of the maximum electrothermal velocity, which is validated by comparing the manipulation velocity and the corresponding offset positions with the velocity profile owing to electrothermal flow (Figure 3b). As expected, the maximum manipulation offset *x*_max_ corresponds with the position of peak electrothermal velocity, and both the sets of data points overlap with each other.

The greater the peak manipulation velocity, the faster the nanotubes are moved to a desired area on the REP chip. Therefore, it is desirable to control the peak manipulation velocity to improve overall MWCNT throughput. Changing the electric field strength can vary the peak manipulation velocity because of the dependence of electrothermal body force on the field strength^33,37^. Figure 3b demonstrates that the measured velocity profile for the electric field of 140 kV m^−1^ is higher than the velocity values for 100 kV m^−1^.

### Trapping and manipulation of an ensemble of MWCNTs

**Figure 4:**
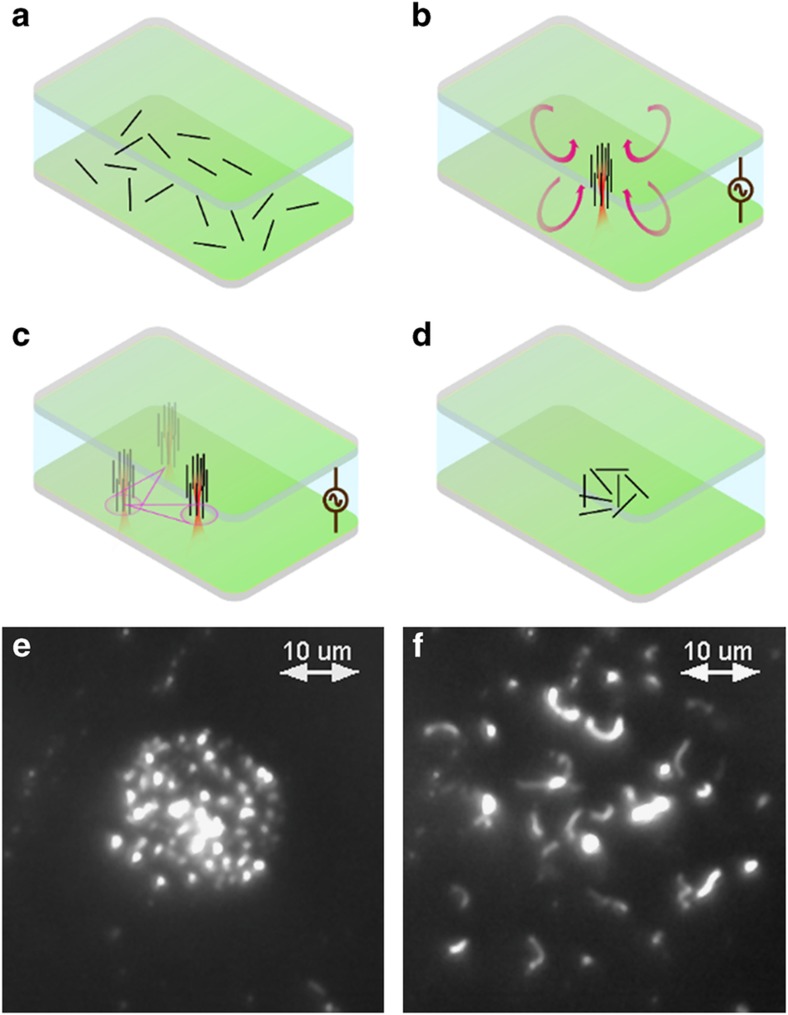
REP trapping and manipulation of multiple MWCNTs. (**a**) MWCNTs in the absence of an electric field and laser. (**b**) Application of the AC electric field (140 kV m^−1^, 15 kHz) and the 20-mW laser beam results in trapping of nanotubes at the center of the electrothermal vortex. (**c**) The trapped nanotubes are manipulated in a similar manner as a single nanotube. (**d**) Removal of the laser and the electric field releases the trapped nanotubes. (**e**) A dark-field image of vertically oriented trapped nanotubes. (**f**) An image of the nanotubes after removing the electric field and the laser.

Trapping a single nanotube provides the ability to control and arrange individual nanotubes on a substrate. However, conventional applications, such as chemical and biosensors, use an ensemble of nanotubes to improve the signal-to-noise ratio^4,8^. Therefore, in addition to single-nanotube trapping, parallel trapping of MWCNTs in a REP trap must be investigated. A concentrated sample of MWCNTs (1.6×10^8^ particles per ml) was used to demonstrate the trapping of multiple nanotubes. Figure 4 and Movie M2 in the Supplementary Information show the trapping process. The application of an AC electric field (140 kV m^−1^, 15 kHz) induced an electric dipole around the nanotubes. These polarized nanotubes were vertically aligned by the electric-field-induced torque. Within a few seconds after vertical alignment, the nanotubes were self-arranged because of an in-plane dipole–dipole repulsive force from each other^43,44^. The implementation of a laser beam accumulated them (Figures 4a, b, and e) against the dipole–dipole repulsion^30^. Similar to manipulating a single nanotube, the trapped cluster of nanotubes was translated on the electrode surface by translating the REP chip (Figure 4c). Removing the laser beam resulted in an increase in the internanotube spacing owing to the dipole–dipole repulsion. The nanotubes were released from their vertical orientation by removing the electric field (Figures 4d and f).

### Electrophoretic deposition of the MWCNTs

**Figure 5:**
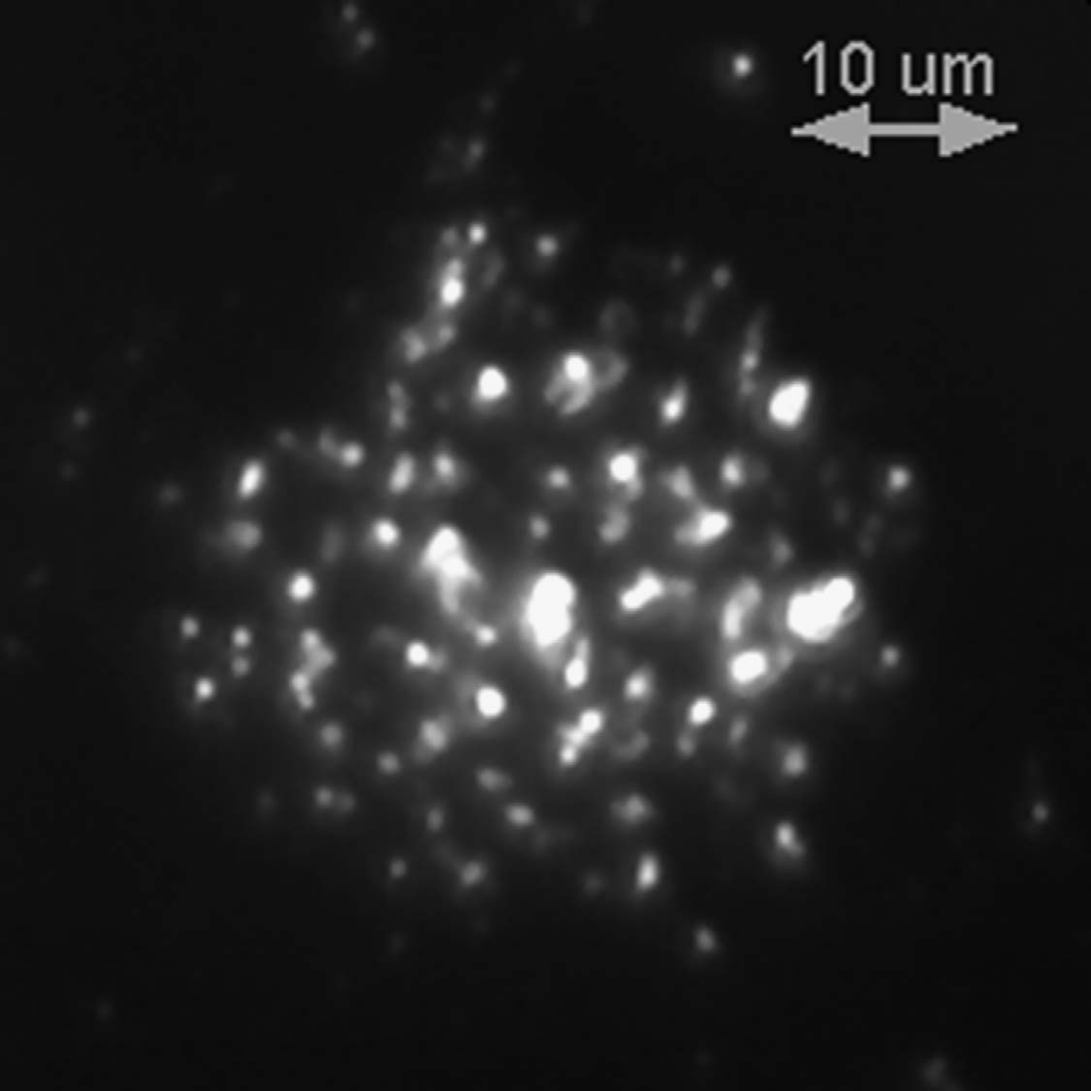
A dark-field image of the electrophoretically deposited vertical MWCNTs. After deposition, the nanotubes retained their vertical orientation in solution without the continuous application of an electric field.

The trapped nanotubes can be permanently fixed at any desired location onto an electrode surface. In conventional electrophoretic deposition, nanotubes are uniformly deposited over the electrode. Alternatively, by using REP, the density of the deposited nanotubes can be varied over the substrate, allowing us to pattern these nanotubes. This patterning was achieved by increasing the electric field strength to 200 kV m^−1^ and applying a direct current (DC) offset of 12 kV m^−1^. Figure 5 and Movie M3 in the Supplementary Information show this deposition process. During reversible trapping (Figure 4f), the nanotubes lost their vertical orientation upon removal of the electric field. In contrast, after performing the electrophoretic deposition process, the vertically aligned MWCNTs remained adhered to the substrate, even after the laser beam and electric field were disabled.

### Controlling the number of nanotubes in a REP trap

**Figure 6:**
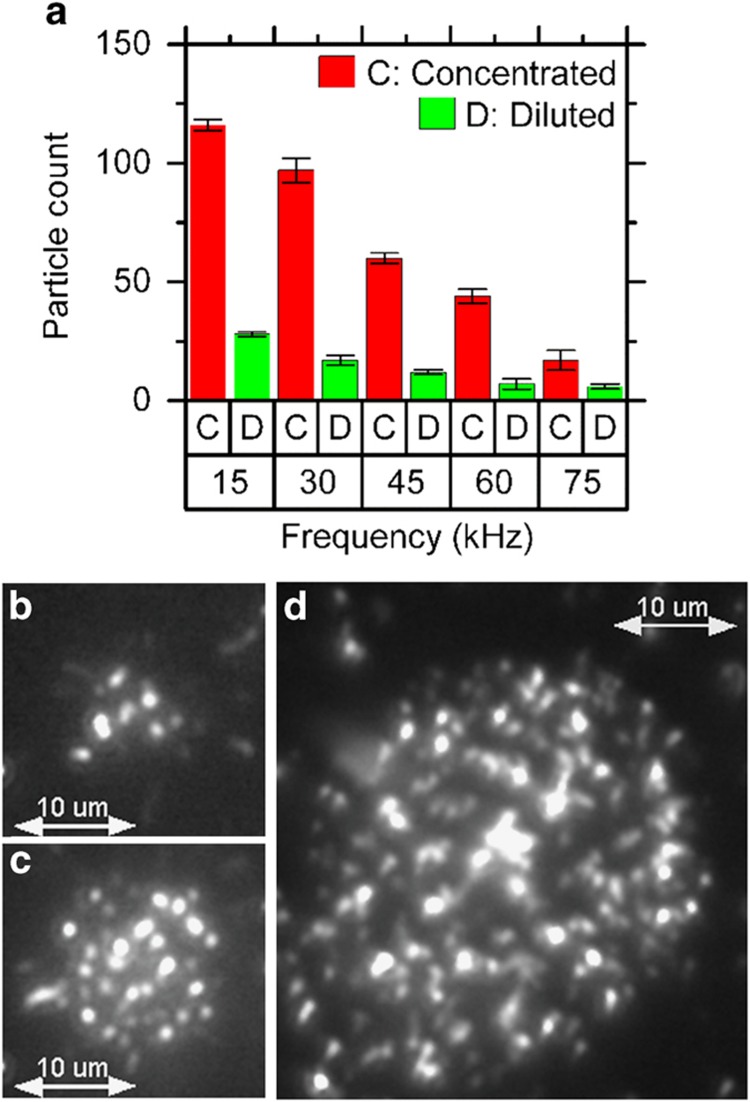
Controlling the number of nanotubes in a REP trap. (**a**) The number of nanotubes in a REP trap as a function of AC frequency for concentrated (2.3×10^8^ particles per ml) and diluted (7.8×10^7^ particles per ml) MWCNT dispersions. (**b** and **c**) Dark-field images of the trapped nanotubes for the dilute dispersion at AC frequencies of 75 and 15 kHz, respectively. (**d**) An image of trapped nanotubes for the concentrated dispersion at an AC frequency of 15 kHz. The number of trapped nanotubes increases with decreasing frequency and increasing nanotube concentration.

Control over the number of nanotubes in a REP trap is governed by two parameters (Figure 6a): (1) the electrical frequency and (2) the concentration of dispersed nanotubes. With regard to (1), as the AC frequency was lowered from 75 kHz to 15 kHz, the number of nanotubes in a REP trap increased. Figures 6b and c show dark-field images of the cluster of nanotubes at AC frequencies of 15 and 75 kHz, respectively. From these images, the increase in the number of nanotubes at low-AC frequency is clearly observable. This increase can be attributed to improved particle–electrode interactions at the lower AC frequencies^35^. With respect to (2), Figures 6c and d show an REP cluster for both the diluted (7.8×10^7^ CNTs per ml) and concentrated (2.3×10^8^ CNTs per ml) samples at an AC frequency of 15 kHz. The number of trapped nanotubes was quadrupled by a threefold increase in the concentration of nanotubes.

## Outlook

The ability to position purified carbon nanotubes at well-defined locations on a substrate remains a challenge for the fabrication of CNT-driven electronics and sensors. In this study, we presented a post-growth, bottom–up technique, called REP, for the trapping and deposition of CNTs on an electrode surface. By using laser-induced electrothermal flow and particle–electrode interactions, REP can trap a range of CNTs, from a single CNT to an ensemble of CNTs, on an electrode surface. The trapped nanotubes are translated by moving the laser beam, and the CNTs are deposited at a desired region by applying an asymmetrical AC electric field with a net DC component. Using the Equipartition theorem, the stiffness of the REP trap in the *x* direction was determined to be 43.3±0.9 fN μm^−1^. In addition, we demonstrated that the peak manipulation speed of a trapped nanotube is dependent on the electrothermal flow velocity, which is changed by varying the AC electric field. REP offers various advantages in patterning CNTs on an electrode surface. REP can be applied to purified CNT samples, and the number of CNTs in a REP trap can be dynamically tuned by either changing the AC frequency or tuning the concentration of the suspended nanotubes. As REP utilizes electric-field-induced effects, we expect to observe similar results for the trapping and deposition of various nanowires.
