# Supplementary material for: Dynamic optoelectric trapping and deposition of multiwalled carbon nanotubes
Source: Microsyst Nanoeng. 2016 Mar 24;2:16005. doi: 10.1038/micronano.2016.5 (PMC6444741; doi:10.1038/micronano.2016.5)
Supplement: Supplementary Information [file micronano20165-s1.pdf]

Supplementary Information

Dynamic optoelectric trapping and deposition of multiwalled carbon nanotubes

Avanish Mishra[1], Katherine Clayton[1], Vanessa Velasco[2], Stuart J. Williams[2] and Steven T. Wereley[1]

Figure S1a shows a microscope image of the MWCNT in the trap, and Figure S1b shows the diffusion path of the tube over a period of four seconds.

Position sensing of a MWCNT in a Rapid Electrokinetic Patterning trap was performed by recording the images of an MWCNT with a CCD camera at 10 FPS. The captured images were spatially calibrated with a microruler (1 px = 0.185 μm). There are various algorithms available that can track centroid of particles with sub-pixel accuracy[1]. We used ImageJ for tracking the x and y coordinates of the centroid of the imaged MWCNT cross-section. From this data, equilibrium position of the trapped MWCNT (x_{eq}, y_{eq}) and position variance in x and y directions ($\langle x^2\rangle$, $\langle y^2\rangle$) were calculated by Equations S1a and S1b, and Equations S2a and S2b[2]. Uncertainty in values of position variance was calculated by conducting uncertainty analysis on Equations S2a and S2b.

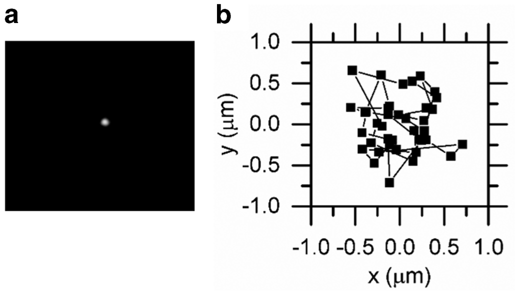

Figure S1 (**a**) Darkfield image of a vertically oriented MWCNT in the REP trap and (**b**) diffusion path of the tube.

Assuming subpixel resolution, each position measurement has an uncertainty of 0.1 px (= 0.018 μm) which can be utilized to calculate total uncertainty in variance. Readers are referred to the Sarshar *et al.*[1] and Raffel *et al.*[3] for more discussion on the video microscopy based position sensing and the subpixel estimation, respectively.

$$x_{eq} = \frac{1}{N}\sum_{i=1}^{N} x_i \tag{S1a}$$

$$y_{eq} = \frac{1}{N}\sum_{i=1}^{N} y_i \tag{S1b}$$

$$\langle x^2\rangle = \frac{1}{N}\sum_{i=1}^{N} \left(x_i - x_{eq}\right)^2 \tag{S2a}$$

$$\langle y^2\rangle = \frac{1}{N}\sum_{i=1}^{N} \left(y_i - y_{eq}\right)^2 \tag{S2b}$$

REFERENCES

1 Sarshar M, Wong WT, Anvari B. Comparative study of methods to calibrate the stiffness of a single-beam gradient-force optical tweezers over various laser trapping powers. *Journal of Biomedical Optics* 2014; **19**: 1150012014.

2 Jones P, Maragó OM, Volpe G. Optical Tweezers: Principles and Applications. Cambridge University Press, 2015.

3 Raffel M, Willert CE, Wereley ST, Kompenhans J. Particle Image Velocimetry: A Practical Guide. Springer Science & Business Media, 2007.

[1]Birck Nanotechnology Center, School of Mechanical Engineering, Purdue University, West Lafayette, IN 47907, USA and [2]Department of Mechanical Engineering, University of Louisville, KY, USA.
Correspondence: Steven T. Wereley (wereley@purdue.edu)
